# Electrospun PLLA Nanofiber Scaffolds and Their Use in Combination with BMP-2 for Reconstruction of Bone Defects

**DOI:** 10.1371/journal.pone.0025462

**Published:** 2011-09-28

**Authors:** Markus D. Schofer, Philip P. Roessler, Jan Schaefer, Christina Theisen, Sonja Schlimme, Johannes T. Heverhagen, Maximilian Voelker, Roland Dersch, Seema Agarwal, Susanne Fuchs-Winkelmann, Jürgen R. J. Paletta

**Affiliations:** 1 Department of Orthopedics and Rheumatology, University Hospital Marburg, Marburg, Germany; 2 Department of Diagnostic Radiology, University Hospital Marburg, Marburg, Germany; 3 Department of Macromolecular Chemistry, Philipps-University Marburg, Marburg, Germany; University of Minho, Portugal

## Abstract

**Introduction:**

Adequate migration and differentiation of mesenchymal stem cells is essential for regeneration of large bone defects. To achieve this, modern graft materials are becoming increasingly important. Among them, electrospun nanofiber scaffolds are a promising approach, because of their high physical porosity and potential to mimic the extracellular matrix (ECM).

**Materials and Methods:**

The objective of the present study was to examine the impact of electrospun PLLA nanofiber scaffolds on bone formation *in vivo*, using a critical size rat calvarial defect model. In addition we analyzed whether direct incorporation of bone morphogenetic protein 2 (BMP-2) into nanofibers could enhance the osteoinductivity of the scaffolds. Two critical size calvarial defects (5 mm) were created in the parietal bones of adult male Sprague-Dawley rats. Defects were either (1) left unfilled, or treated with (2) bovine spongiosa, (3) PLLA scaffolds alone or (4) PLLA/BMP-2 scaffolds. Cranial CT-scans were taken at fixed intervals *in vivo*. Specimens obtained after euthanasia were processed for histology, histomorphometry and immunostaining (Osteocalcin, BMP-2 and Smad5).

**Results:**

PLLA scaffolds were well colonized with cells after implantation, but only showed marginal ossification. PLLA/BMP-2 scaffolds showed much better bone regeneration and several ossification foci were observed throughout the defect. PLLA/BMP-2 scaffolds also stimulated significantly faster bone regeneration during the first eight weeks compared to bovine spongiosa. However, no significant differences between these two scaffolds could be observed after twelve weeks. Expression of osteogenic marker proteins in PLLA/BMP-2 scaffolds continuously increased throughout the observation period. After twelve weeks osteocalcin, BMP-2 and Smad5 were all significantly higher in the PLLA/BMP-2 group than in all other groups.

**Conclusion:**

Electrospun PLLA nanofibers facilitate colonization of bone defects, while their use in combination with BMP-2 also increases bone regeneration *in vivo* and thus combines osteoconductivity of the scaffold with the ability to maintain an adequate osteogenic stimulus.

## Introduction

Surgical reconstruction of bone defects after injury or tumor resection frequently requires the use of graft material. Autologous bone grafts are a widely accepted standard of bone repair and regeneration. Although there are many advantages to the use of bone grafts, major drawbacks such as donor site morbidity and restricted availability affect approximately 10% of patients in clinical practice [1], [2]. To overcome these drawbacks, artificial bone grafts based on synthetic biomaterials such as metals, polymers, porous ceramics, hydroxyapatite, collagen sponges or hydrogels as well as several composites have been developed recently [2]–[5]. Moreover, to engineer an effective bone graft material, substances that are capable of triggering osteogenesis such as growth factors have to be included [6]. Therefore a scaffold should ideally function as a carrier for growth factors as well as cells [7], [8]. To support the latter, a scaffold must be three-dimensional and porous, mimicking the extracellular matrix (ECM) produced by healthy bone [9]. Considering this aspect, scaffolds based on nanofibers offer great advantages [10], [11] and the nano-fibrous architecture may serve as a superior scaffold compared to solid-walled architecture for the promotion of osteoblast differentiation and biomineralization [12]. Nanofibers can be obtained by several methods including self-assembly [13]–[18], thermally induced liquid–liquid phase separation for the formation of nanofibrous foam materials [19], [20] or carbon dioxide laser supersonic drawing [21] and electrospinning. Each approach has unique advantages, lending itself to development as a scaffolding system. Self-assembly for example can generate small diameter nanofibers at the lowest end of the size-range of natural extracellular matrix collagens. These scaffolds can support growth and differentiation of MSC [18], [22] *in vitro* as well as *in vivo* [17] and may serve as a drug delivery system [23]. Phase separation, on the other hand, generates nanofibers in the same range as natural extracellular matrix collagens and allows for the design of macropore structures. Electrospinning is one of the most promising methods of producing continuous fibers on a large scale. Although the original method was first published in 1934 [24], the technique was established in the early 1990s [25]–[27]. Simplified, the process utilizes an electric field in order to charge a viscous polymer solution. As a consequence electrostatic force draws the fluid from the developing Taylor cone into a liquid jet. Due to various interactions between electric field and the charged jet [26], bending instability produces a spiral shaped trajectory. This process is accompanied by solvent evaporation resulting in formation of solid (nano-)fibers deposited on the collector electrode as a non-woven mat. These fiber mats can be generated by electrospinning from a large variety of polymers which have been analyzed with respect to their possible use in tissue engineering applications [3] using fibroblasts [28] tenocytes [29] neural stem cells [30], MSC [31] or osteoblast like cell lines [32].

Whether or not a polymer can be electrospun into nanofibers depends on a variety of factors including voltage, conductivity of the solution, and entanglement density of the polymer, that in their turn depend on its chemical nature and molecular weight, the solvent and concentration used and on environmental conditions [33], [34]. Nevertheless to date more than 100 polymers have been used to produce nanofibers [34]. With respect to tissue engineering, among the polymers tested, biopolymers or biocompatible chemosynthetic polymers are of the greatest interest. Among these, poly-L-lactic acid (PLLA) plays an important role due to its biocompatibility, biodegradability and FDA approval which allows its use in bone reconstructive surgery [35], [36]. As reported earlier, PLLA can easily be electrospun to form a 3D non-woven network [37], [38]. Furthermore, synthetic nanofibers may exhibit certain properties similar to natural collagen fibers and thus may serve as superior scaffolding compared to solid-walled materials in promoting cell migration, differentiation and subsequent biomineralization [12]. Earlier *in vitro* experiments indicated that stem cells grow well on PLLA nanofiber scaffolds. Nevertheless the presence of PLLA nanofibers resulted in a down-regulation of genes associated with the osteoblast linage [39] which can be overcome by combination of the nanofibers with collagen, [39], RGD sequences [39], [40] or BMP-2 [41]. BMP-2, which has been shown to promote osteoblast activity [42] and has been successfully applied in the reconstruction of bone defects in a number of clinical studies [43]–[45], can be incorporated into nanofiber scaffolds in a bioactive form by electrospinning. Although there are some reports that the structural integrity of BMP-2 [46] as well as its bioactivity [47] might be influenced by electrospinning, bioactivity is retained *in vitro* [41].

Srouji et al. recently reported the use of a core-shell nanotube system for the release of BMP-2 *in vitro*, also demonstrating a possible *in vivo* application method [48]. Fu et al. used electrospun PLGA/HAp nanofibers as a delivery system for BMP-2 and observed good osteoinductive activity [47].

To our knowledge, no *in vivo* data are available analyzing the effect of electrospun PLLA nanofiber scaffolds on bone formation in a time dependent manner. Therefore the aims of this study were to characterize the influence of PLLA nanofibers on bone formation *in vivo* and to analyze whether BMP-2 enhances bone healing when incorporated into PLLA nanofiber scaffolds by electrospinning.

## Materials and Methods

### Fabrication of nanofibers

The preparation of PLLA nanofibers by electrospinning was performed under aseptic conditions and has been previously reported [31], [39]. Briefly, a 4% (w/w) PLLA (Resomer L210, Boehringer, Ingelheim, Germany) solution dissolved in dichloromethane was prepared at room temperature by stirring overnight until a homogeneous solution was obtained. The spinning process was performed at a flow rate of 14 µL/min with an applied voltage of 20–30 kV and an electrode distance of 15 cm. In order to incorporate BMP-2 into the nanofibers, 25 µg lyophilized rhBMP-2 (Reliatech, Braunschweig, Germany) were dissolved in 125 µL 50 mM acetic acid and stabilized by the addition of 25 µL fetal calf serum (FCS). This mixture was emulsified in 2.5 mL of a 4% PLLA-dichloromethane solution. Samples of non-woven nanofibers (approximately 1 mm in thickness) were collected on a sterile aluminum plate with an area of 3600 mm^2^ and trimmed to sizes of 25 mm^2^ immediately before implantation. Based on the initial conditions, BMP-2 concentration was about 6.94 ng/µL and thus a single implant contained approximately 174 ng BMP-2. Further characterization and physical properties of PLLA and PLLA/BMP-2 scaffolds have previously been described in detail [31].

### Animals

One hundred twenty five-month-old Sprague-Dawley rats (Harlan Winkelmann, Borchen, Germany) were used in the experiment. The animals were kept in individual plastic cages (Macrolon Type III) in a room maintained at a constant temperature of 22.1°C, with a 12 h light/dark cycle. They had free access to drinking water and standard laboratory pellets (LASQCdiet® Rod16 Rad, LASVendi, Soest, Germany). All experiments were carried out in strict accordance with the recommendations in the Guide for the Care and Use of Laboratory Animals of the NIH and approved by the local Animal Ethics Committee Regierungspräsidium Giessen under reference number V 54 – 19 c 20-15 (1) MR 20/21- Nr. 18/2008.

### Surgery

Animals were divided into four groups of 30 rats prior to surgery. Bilateral full thickness critical size calvarial defects were created in order to double the defect number and to spare the sagittal sinus. Both defects were filled with one of the following materials, dividing the population into four groups: (1) left unfilled as a negative control, (2) press-fit bovine spongiosa implant as a positive control (Tutobone®, Tutogen, Neunkirchen am Brand, Germany), (3) PLLA nanofiber scaffolds or, (4) PLLA/BMP-2 nanofiber scaffolds. Ten animals per group were sacrificed after a healing time of 4, 8, or 12 weeks.

Surgery was performed under general anesthesia by weight-adjusted intraperitoneal injection of xylazine 2% (Rompun®, 10 mg/kg body weight, Bayer Animal Health, Leverkusen, Germany) and ketamine hydrochloride (Ketamin WDT, 100 mg/kg body weight, WDT, Garbsen, Germany). The dorsal part of the cranium was shaved and aseptically prepared with phenoxyethanol (Octenisept®, Schülke & Mayr, Norderstedt, Germany). An approximately 20 mm long sagittal incision was made to include skin and muscle. The periosteum was reflected and trimmed exposing the parietal bones on both sides. Two bilateral 5 mm full thickness critical size defects (CSD) were created using a trephine bur (No. 229.040, Meisinger, Düsseldorf, Germany) and carefully positioned to leave sufficient normal bone surrounding the defects. Constant irrigation with sterile physiological saline solution was applied to prevent overheating of the bone margins. After implantation of the appropriate material according to group, the site was closed by suturing the overlying tissue and skin (Vicryl rapide 3-0, Ethicon, Norderstedt, Germany). All operations were carried out by an experienced surgeon (MDS). To prevent wound infection each rat received a subcutaneous injection of 2 mL ampicillin/sulbactam (A/S Kabi, 0.125 mL/kg body weight, 200,000 I.U./mL, Fresenius Kabi, Bad Homburg v. d. H., Germany).

### Dual-source CCT

Radiographic evaluation was performed 4, 8 and 12 weeks after surgery using cranial computed tomography (CCT) imaging (Somatom Definition, Siemens Medical Systems, Erlangen, Germany) with a resolution of 0.3 mm. All animals were anesthetized as described above for the duration of the CT-scans. Images were transferred to an image analysis workstation (Leonardo, Siemens Medical Systems, Erlangen, Germany) for evaluation. In order to analyze bone tissue repair, the radiological density was measured by placing a region of interest (ROI) of the same size as the original defect over each data set. Bone density was measured in Hounsfield units (HU).

### Harvesting of tissue and sectioning of test specimens

Animals were sacrificed by CO_2_-asphyxiation. Previously blood was collected by cardiac puncture using a serum-gel tube and screened for signs of inflammation (C-reactive protein) to evaluate the animals' postoperative systemic condition. The defect sites were removed together with a small amount of surrounding bone, skin and soft connective tissue. These samples were immediately fixed in 4% buffered formalin for three days and then decalcified in an EDTA-solution (Osteosoft®, Merck, Darmstadt, Germany) over a period of 18 days. After trimming the bone specimens with a precision saw they were dehydrated in graded alcohol solution and cedar wood oil and embedded in paraffin. Sections were cut at 5 µm with a 40° stainless-steel blade on a rotation microtome (RM2055, Leica Microsystems, Bensheim, Germany).

### Histological and immunohistological staining

Histological staining was performed with Hematoxylin-Eosin (HE) (Merck Chemicals, Darmstadt, Germany) and Masson Goldner (MG) (Merck Chemicals) formulations according to standard protocols.

For immunohistological staining the sections were rehydrated and endogenous peroxidase activity quenched with a 4% hydrogen peroxide solution. They were blocked with normal horse serum (Santa Cruz, Heidelberg, Germany) and incubated overnight with a polyclonal IgG antibody against either osteocalcin, diluted 1∶50 (FL-100, Santa Cruz), BMP-2, diluted 1∶25 (N-14, Santa Cruz) or Smad5, diluted 1∶25 (D-20, Santa Cruz). Sections were then incubated with a biotinylated secondary antibody (Santa Cruz) diluted 1∶50 for 30 min at room temperature. An avidin-biotin-complex detection system coupled with DAB as a chromogen (Santa Cruz) was used to visualize antibody binding after 10 min incubation at room temperature. Finally all sections were counterstained with Gill's hematoxylin solution (Santa Cruz) diluted 1∶2 for 10 sec. Negative controls, incubated without primary antibody, were treated in parallel with each of the previously described staining procedures.

### Histological, immunohistological and histomorphometric analysis

All sections were histologically assessed following standard Hematoxylin-Eosin staining prior to further investigation. Histomorphometric analysis was performed in Masson-Goldner trichrome-stained sections at a primary magnification of 5-fold using a digital microscope (DM5000, Leica Microsystems, Bensheim, Germany) and QUIPS analysis software (Leica Microsystems). Nine images per specimen were captured and assembled into a montage displaying the whole defect. Formation of new bone was calculated in relation to the whole defect area of each section and expressed as a percentage. In the bovine spongiosa implants (positive control) the histologically lighter trabecular areas of the implants were disregarded, in order to quantify the formation of new bone only. Cell counts were performed in five fields per specimen, ranging from one end of the defect to the other, using a primary magnification of 40-fold. Immunohistological evaluation was carried out by selecting four representative regions of interest (ROI) at 20-fold magnification - two regions in the marginal areas of the defect and two in the center – connecting both defect margins together.

### Statistical analysis

Analysis of variance (ANOVA) was used to evaluate the differences between experimental and control groups as well as between different time points in a group. Data are given as means ± standard deviation (SD). The level of significance was set at p<0.05.

## Results

Two animals were lost during surgery due to blood loss. Another animal (PLLA/BMP-2 group) was lost postoperatively due to rapid weight loss. All other animals (n = 117) survived and the implant sites healed well. Animals in groups receiving either bovine spongiosa, PLLA or PLLA/BMP-2 showed firm fixation of the implants on palpation. Groups receiving no implant macroscopically showed formation of a soft membrane of fibrous connective tissue. Serum blood analysis of C-reactive protein (CRP) yielded no signs of infection or inflammation. Levels in the negative control group decreased from 44.56±2.47 mg/L at week four to 19.85±1.68 mg/L after twelve weeks (p≤0.001), in the bovine spongiosa group from 39.52±2.12 mg/L to 18.94±2.15 mg/L (p≤0.001), in the PLLA group from 43.21±8.67 mg/L to 19.38±3.53 mg/L (p≤0.001) and in the PLLA/BMP-2 group from 39.56±2.31 mg/L to 17.84±1.59 mg/L (p≤0.001) with no significant differences between groups. The mean body weights of all animals increased from 236.77±19.34 g to 417.13±46.58 g (p≤0.001) during the three months of the study with no significant differences between groups.

### PLLA exhibits highest colonization rates

**Figure 1:**
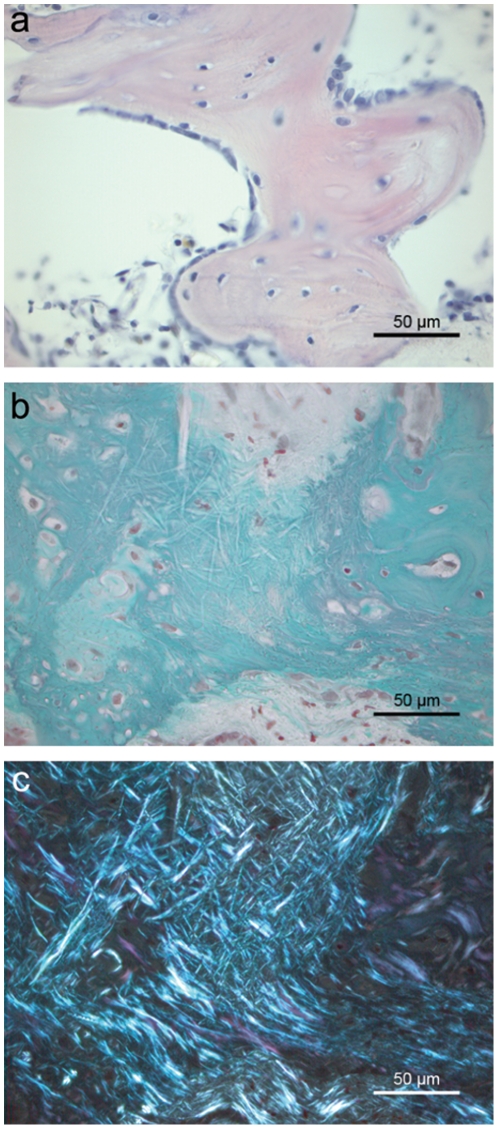
Histological analysis of a defect filled with PLLA/BMP-2. (**a**) New bone ossicle with active osteoblasts on the upper right edge and lining cells on the opposite edge in a defect implanted with PLLA/BMP-2 after 8 weeks (Hematoxylin-Eosin, BF). (**b**) New bone formation in a defect filled with PLLA/BMP-2 after 12 weeks, viewed under visible light, showing the remains of PLLA nanofibers (Masson-Goldner, BF). (**c**) The same micrograph as in ‘b’ under polarized light microscopy, revealing the full extent of bone incorporation through the nanofiber scaffold as well as some loose collagen fibers at the top (Masson-Goldner, POL).

Empty negative control defects (Group 1) did not show any relevant regeneration by histology at any time during the experiment. Instead, a membrane of fibrous connective tissue formed between the two margins of the bony defect. Positive control defects implanted with bovine spongiosa (Group 2) showed a late onset of bone formation, which began slowly after 4 weeks, linked to degradation of the avital implant trabeculae. Most of the implant was resorbed after 12 weeks, resulting in newly-formed bone marrow spaces. Defects implanted with plain PLLA scaffolds (Group 3) were colonized by large numbers of cells, but showed only a small amount of bone formation. Ossification mainly took place in the marginal areas of the defect adjacent to old vital bone. Defects implanted with PLLA/BMP-2 (Group 4) showed an early onset of bone regeneration throughout the whole defect site after 4 weeks (Fig. 1a). Formation of bone marrow spaces and continuous osteointegration at the defect margins could be observed after 8 weeks.

**Figure 2:**
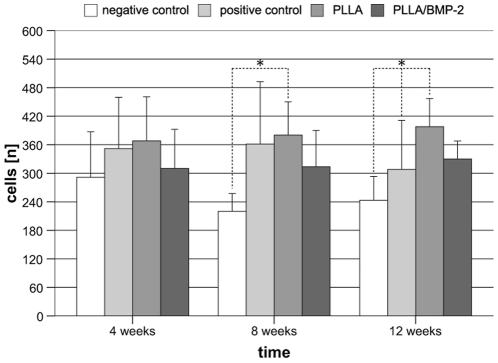
Mean cell densities [n] per implant. Mean cell densities per implant reached the highest levels in the PLLA group. After 8 weeks cell densities in PLLA and bovine spongiosa groups were significantly higher than in the negative control group. After 12 weeks cell densities in the PLLA group were significantly higher than in negative control and bovine spongiosa groups. *p<0.05.

To elucidate whether PLLA nanofiber scaffolds support the formation of cell settlements in a bone defect, cell counts of HE stained sections were performed. As shown in figure 2, cell densities were increased when scaffolds were implanted in the defect compared to negative controls. Significance was reached in the case of PLLA nanofiber scaffolds in comparison to negative control defects (p≤0.001) and positive control defects (p = 0.048) after 12 weeks (Fig. 2). Finally, polarized light microscopy provided evidence that PLLA and PLLA/BMP-2 nanofibers could be incorporated into the newly formed bone without the interposition of connective tissue (Fig. 1b+c). No histological foreign body reaction could be seen with either PLLA or PLLA/BMP-2 implants.

### Bone density gain in PLLA/BMP-2 is higher than in bovine spongiosa

**Figure 3:**
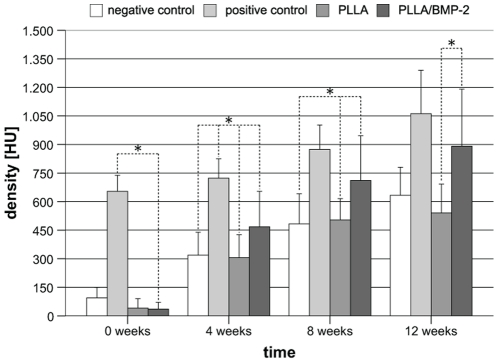
Radiological density [HU] of defect areas as measured by cranial CT-scans. The bovine spongiosa group increased by around 400 HU over the 12 week period, comparable to that of the PLLA group. The PLLA/BMP-2 group in contrast increased by more than 850 HU over the same period. There was no longer any statistically significant difference between bovine spongiosa and PLLA/BMP-2 at weeks 8 and 12. *p<0.05.

**Figure 4:**
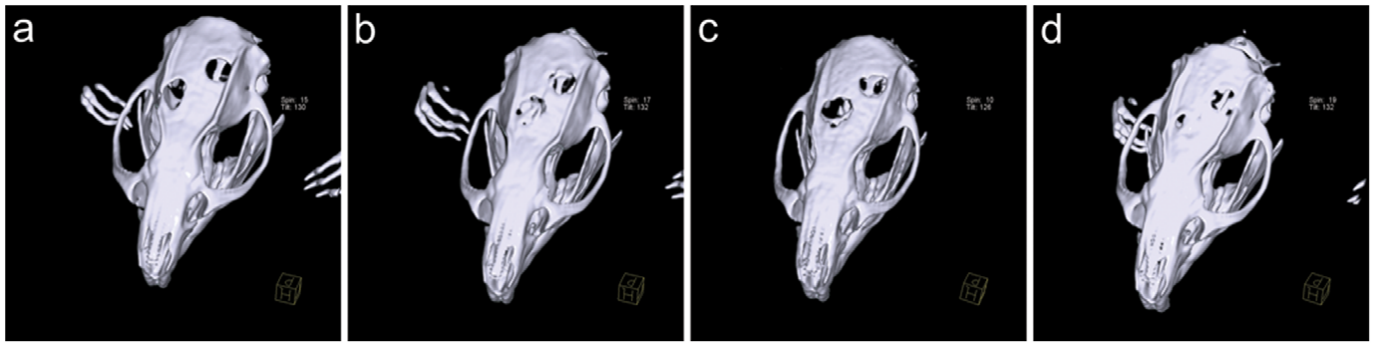
3D reconstructions of cranial CT-scans used for quantification of radiological bone density. (**a**) Negative control, 12 weeks. (**b**) Positive control, 12 weeks. (**c**) PLLA, 12 weeks. (**d**) PLLA/BMP-2, 12 weeks.

When bovine spongiosa was implanted into defects, means of 654±84 HU were detected after 4 weeks, which was significantly higher than PLLA or PLLA/BMP-2 nanofiber scaffolds. (Fig. 3) Within three months, radiological densities increased independent of treatment as demonstrated in figure 3. It is remarkable that this increase was approximately two times higher in animals implanted with PLLA/BMP-2 nanofiber scaffolds compared to the other treatments (856 HU increase with PLLA/BMP-2 versus 499 HU increase with PLLA; 409 HU increase with bovine spongiosa and 538 HU increase in untreated defects). This resulted in higher bone densities as compared to empty defects (p = 0.003 after 4 weeks; p = 0.003 after 8 weeks and p = 0.083 after 12 weeks) as well as blank PLLA nanofiber scaffolds (p≤0.001 after 4 weeks; p = 0.013 after 8 weeks and p = 0.008 after 12 weeks). Furthermore, after 12 weeks there was no longer any statistical difference between the bovine spongiosa group (positive control) and defects treated with PLLA/BMP-2 nanofiber scaffolds (p = 0.666). Figure 4 shows 3D reconstructions of the cranial CT-scans of all four groups after 12 weeks (Fig. 4).

### PLLA/BMP-2 induces early-onset bone formation

**Figure 5:**
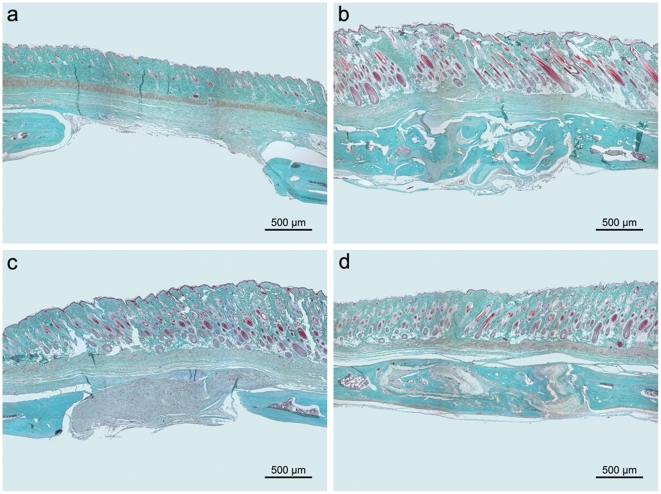
Montages (3×3) used for histomorphometry generated with the Leica QUIPS package. (**a**) Negative control, 12 weeks. (**b**) Positive control, 12 weeks. (**c**) PLLA, 12 weeks. (**d**) PLLA/BMP-2, 12 weeks (Masson-Goldner, BF).

**Figure 6:**
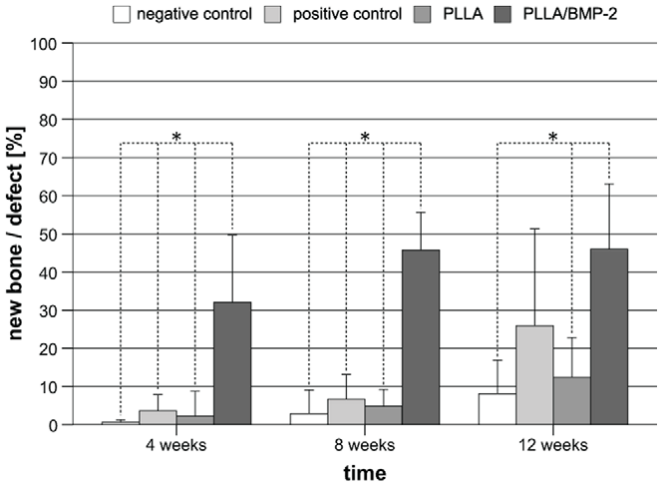
Formation of new bone in relation to the whole defect area [%] as determined by histomorphometry. Defects implanted with PLLA/BMP-2 showed significantly faster bone regeneration than every other group. After 12 weeks, a significant difference could no longer be detected between PLLA/BMP-2 and bovine spongiosa groups, although mean relative bone formation differed by ∼20%. *p<0.05.

The observed increase in radiological bone density was reflected in the formation of hard callus as determined by histomorphometry (Fig. 5). After implantation of PLLA/BMP-2 nanofiber scaffolds approximately 30% of the defect site were filled with hard callus after 4 weeks as shown in figure 6. This was significantly higher than that observed in all other treatments (p≤0.001). During the course of the experiment, callus formation in the PLLA/BMP-2 group increased up to 45% after 12 weeks which was significantly higher than hard callus formation in the negative control group (p≤0.001) and the PLLA group (p = 0.002), but there was no significant difference between the PLLA/BMP-2 group and the bovine spongiosa group after 12 weeks (p = 0.140) (Fig. 6).

### Use in combination with BMP-2 leads to sustained osteoinduction

**Figure 7:**
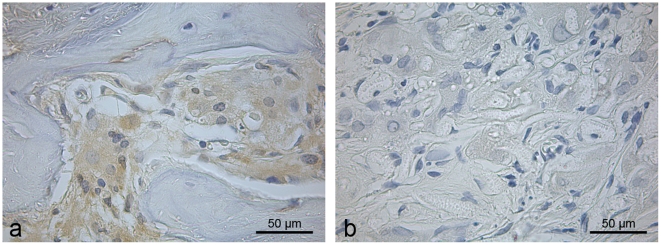
Immunohistological staining for Osteocalcin. (**a**) Osteocalcin-positive cells in an ossicle of new bone adjacent to nanofiber scaffold in a defect implanted with PLLA/BMP-2 after 8 weeks. (**b**) Negative control for osteocalcin from the center of the same defect (DAB-Hematoxylin, BF).

**Figure 8:**
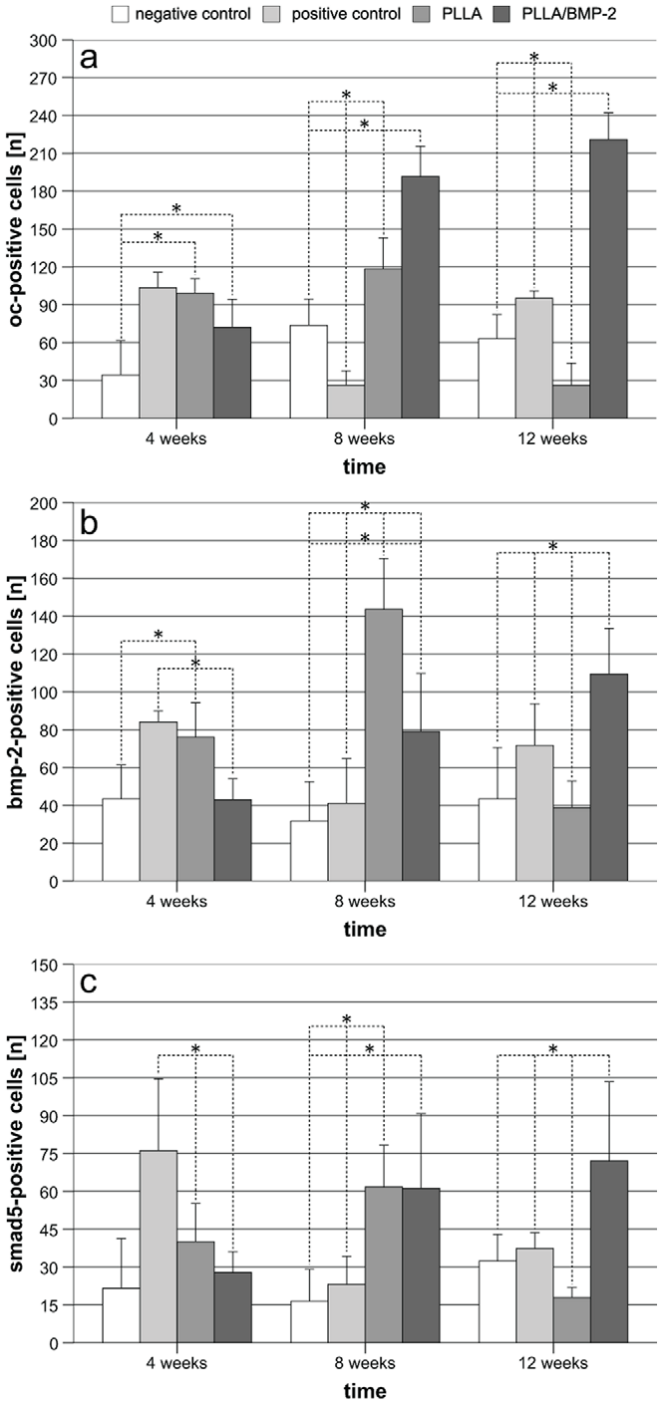
Mean cell densities [n] of Osteocalcin-, BMP-2- and Smad5-positive cells as determined by immunohistochemistry. (**a**) Mean osteocalcin-positive cells [n] as determined by immunostaining. From 8 weeks onwards, differences between the PLLA/BMP-2 group and all other groups are highly significant. *p<0.05 (**b**) Mean BMP-2-positive cells [n] as determined by immunostaining. Expression of BMP-2 increased with a delay of 4 weeks in the PLLA/BMP-2 group compared to osteocalcin. After 12 weeks, BMP-2 levels of the PLLA/BMP-2 group were significantly different from all other groups. *p<0.05 (**c**) Mean Smad5-positive cells [n] as determined by immunostaining. Similar to the expression of BMP-2, the expression of Smad5 reached its maximum after 12 weeks in the PLLA/BMP-2 group. *p<0.05.

Increased hard callus formation in the PLLA/BMP-2 group was accompanied by a constant increase of osteocalcin-positive cells as determined by immunostaining (Fig. 7). PLLA/BMP-2 scaffolds induced significantly higher expression of osteocalcin (p≤0.001) compared to all other implants after 8 and 12 weeks. PLLA scaffolds showed a tendency towards growth at first, but then reached values even lower than the negative group (p = 0.007) after 12 weeks (Fig. 8a).

An increasing number of BMP-2 positive cells could also be observed in the PLLA/BMP-2 group. After 12 weeks the PLLA/BMP-2 group showed a significantly higher expression of BMP-2 than all other groups (negative control and PLLA p≤0.001; positive control p = 0.05). It is remarkable that the expression of BMP-2 in the PLLA group reached a highly significant maximum after 8 weeks (p≤0.001) before decreasing again towards the end of the observation period (Fig. 8b).

With regard to the expression of Smad5, a significant dominance of the positive control group (negative control p≤0.001; PLLA p = 0.025 and PLLA/BMP-2 p = 0.002) could be observed after 4 weeks, but this difference had already disappeared after 8 weeks. Both PLLA and PLLA/BMP-2 groups exhibited equal numbers of Smad5 positive cells after 8 weeks, higher than both negative and positive control groups. After 12 weeks the expression of Smad5 was significantly higher in the PLLA/BMP-2 group than in all other groups (negative control p = 0.003; positive control p = 0.011 and PLLA p≤0.001) (Fig. 8c).

## Discussion

With regard to the reconstruction of critical size calvarial defects in the rat, three main options have been described in the literature: bare bridging with xenogenic [49] or allogenic [50] substances, implants combined with growth factors [51]–[53] or gene therapy using modified MSCs [54], [55]. Synthetic xenogenic 3D implants may have certain different characteristics ranging from different initial materials to varying 3D structure.

Achieving adequate osteointegration of a scaffold seems to be more of a challenge than incorporation of growth factors, although there may be a link between these two properties [56]. As Woo et al. recently showed, 3D nanofibrous PLLA scaffolds are superior to solid walled PLLA scaffolds of equal porosity in respect to bone regeneration *in vivo*. This effect was traced back to the fact that nanofiber scaffolds may mimic the fibrous morphology of type I collagen and therefore of bone ECM [57]. Although nanofiber scaffolds undergo faster degradation *in vitro* compared to solid walled scaffolds, it is not yet clear if this effect also contributes to their superior properties *in vivo* [58]. To make use of the positive effects mentioned above and to facilitate cell migration, PLLA nanofiber scaffolds were implanted into critical size defects. These scaffolds were colonized by cells resulting in significantly higher cell densities as compared to empty defects, indicating that the nanofiber scaffold forms a stable matrix for filling large bone defects. This was confirmed by polarized light microscopy demonstrating a nanofiber fiber network within the defect over a period of 3 months. However, based on *in vitro* data obtained by other researchers [59], it can be assumed that the mechanical stability of the scaffold decreases over time. In this case, the onset of new bone formation has to occur early in order to compensate for this effect. In our study, PLLA nanofibers alone had no impact on the formation of new bone as demonstrated by histomorphometry, immunostaining or CT scans. These findings correspond well to earlier *in vitro* experiments indicating that plain PLLA nanofiber scaffolds have a positive effect on cell density, but also result in down-regulation of genes associated with the osteoblast lineage [31], [60]. This delay in osteoblast differentiation can be overcome by application of BMP-2, which has been evaluated in conjunction with a number of carrier substances [2]–[5].

As each carrier has an influence on growth factor delivery, physicochemical and biological properties of its initial material are very important. Most carriers loaded with BMP-2 show an early burst of BMP-2 release with a reduction of retained BMP-2 to less than 10% within the first 5 days [61].

When incorporated in core shell fibers, the release pattern of BMP-2 can be modulated by variations in the polymer-ratios between polycaprolactone and polyethyleneoxide [48]. This release can be prolonged by direct incorporation of BMP-2 into Poly(lactide-cp-glycolide)/hydroxylapatite composite nanofibers [47]. When electrospun into nanofibers the incorporated rhBMP-2 retains its structural integrity with respect to size and allotment of α-helices, β-sheets, helix-turn-helices and β-antiparallel structures, although stabilization seemed to be necessary in some way [46], [47], [62]. Therefore the core shell fibers as well as the PLGA/HAp nanofibers are able to induce osteoblast differentiation as well as formation of new bone. Similar results can be obtained when hMSC are cultured on PLLA/BMP-2 nanofiber scaffolds, which has been found to induce gene expression of osteocalcin and collagen as well as alkaline phosphatase *in vitro* [41].

When implanted into a critical size defect, a significant increase of hard callus formation could be observed as early as 4 weeks, accompanied by an increase in bone density as determined by CT scans. Taken together with the rapid increase in osteocalcin-positive cells after 8 weeks and subsequent up-regulation of BMP-2 and Smad5 after 12 weeks, these results indicate that the incorporated rhBMP-2 should be considered bioactive within the PLLA nanofiber scaffolds *in vivo*. Therefore the actual *in vivo* data, together with our *in vitro* data published previously, demonstrate that PLLA nanofibers can be successfully modified by direct incorporation of BMP-2. Implant degradation of PLLA nanofibers did not seem to affect the formation of new bone in the PLLA/BMP-2 group. Moreover, the fibers were incorporated into the newly-formed bone. Therefore the assumed degradation of the PLLA nanofibers and the resulting decrease in mechanical stability (Paletta et al. 2010) will be compensated by formation of new bone. However in this study a non-weight-bearing model was used. With respect to weight bearing bones, the initial stability of nanofiber scaffolds alone is not sufficient. Here, additional fixation seems to be necessary until the new bone bridges the defect and fibers are incorporated into the bone. It is our opinion that this incorporation screens the material from the immunological system. This is supported by the finding that no signs of immunologic reaction were observed, either histologically or by blood analysis.

These findings lead to the assumption that PLLA/BMP-2 nanofiber scaffolds can overcome these adverse effects either because BMP-2 is incorporated bioactively and preserved on the surface throughout the whole observation period, or because of the low total amount of PLLA due to the scaffold's high porosity.

Some other experimental models also make use of BMP-2 as a growth factor as it is well known to promote bone healing. Similar results on the efficacy of BMP-2 were observed in a study by Patel et al. comparing different types of rhBMP-2-carrying implants [51], as well as Patterson et al., who tested hyaluronic acid hydrogels carrying BMP-2 [49] and Young et al., who used a similar system [63]. To achieve sufficient osteogenesis or defect closure however, relatively high concentrations of BMP-2 were needed in either defect-based [49], [64], [65] or ectopic models [61]. Electrospun nanofibers loaded with rhBMP-2 represent a viable alternative here because of their 3D ECM-like structure, their high physical porosity and their ability to incorporate a bioactive growth factor which may help to reduce the concentration of such factors required.

### Conclusion

The aims of this study were to evaluate the influence of three-dimensional PLLA nanofiber scaffolds on bone formation *in vivo* and to analyze whether incorporated BMP-2 could enhance their efficacy. PLLA nanofiber scaffolds were shown to facilitate cell immigration and thus to achieve high cell densities. However they lacked adequate osteogenic stimuli to allow further differentiation of those cells. The incorporation of rhBMP-2 into PLLA nanofibers could overcome this problem. Hence PLLA/BMP-2 implants were able to close critical size calvarial defects within 8 weeks. Increased expression of osteocalcin, BMP-2 and Smad5 suggests a subsequent activation of the osteoblast lineage. Therefore PLLA/BMP-2 nanofiber scaffolds combine a suitable matrix for cell migration with an osteoinductive stimulus.
